# Gradient boosting for Parkinson’s disease diagnosis from voice recordings

**DOI:** 10.1186/s12911-020-01250-7

**Published:** 2020-09-15

**Authors:** Ibrahim Karabayir, Samuel M. Goldman, Suguna Pappu, Oguz Akbilgic

**Affiliations:** 1 Parkinson School of Health Sciences and Public Health, Loyola University Chicago, 2160 S 1st Street, CTRE #127, Maywood, IL 60153 USA; 2 Kirklareli University, Kirklareli, Turkey; 3 School of Medicine, University of California San Francisco, San Francisco, CA USA; 4 Stritch School of Medicine, Loyola University Chicago, Maywood, IL USA

**Keywords:** Parkinson’s disease, Gradient boosting, Machine learning, Artificial intelligence, Speech test

## Abstract

**Background:**

Parkinson’s Disease (PD) is a clinically diagnosed neurodegenerative disorder that affects both motor and non-motor neural circuits. Speech deterioration (hypokinetic dysarthria) is a common symptom, which often presents early in the disease course. Machine learning can help movement disorders specialists improve their diagnostic accuracy using non-invasive and inexpensive voice recordings.

**Method:**

We used “Parkinson Dataset with Replicated Acoustic Features Data Set” from the UCI-Machine Learning repository. The dataset included 44 speech-test based acoustic features from patients with PD and controls. We analyzed the data using various machine learning algorithms including Light and Extreme Gradient Boosting, Random Forest, Support Vector Machines, K-nearest neighborhood, Least Absolute Shrinkage and Selection Operator Regression, as well as logistic regression. We also implemented a variable importance analysis to identify important variables classifying patients with PD.

**Results:**

The cohort included a total of 80 subjects: 40 patients with PD (55% men) and 40 controls (67.5% men). Disease duration was 5 years or less for all subjects, with a mean Unified Parkinson’s Disease Rating Scale (UPDRS) score of 19.6 (SD 8.1), and none were taking PD medication. The mean age for PD subjects and controls was 69.6 (SD 7.8) and 66.4 (SD 8.4), respectively. Our best-performing model used Light Gradient Boosting to provide an AUC of 0.951 with 95% confidence interval 0.946–0.955 in 4-fold cross validation using only seven acoustic features.

**Conclusions:**

Machine learning can accurately detect Parkinson’s disease using an inexpensive and non-invasive voice recording. Light Gradient Boosting outperformed other machine learning algorithms. Such approaches could be used to inexpensively screen large patient populations for Parkinson’s disease.

## Background

Parkinson ‘s disease (PD) is a neurodegenerative disorder of largely unknown cause [1]. After Alzheimer’s disease, it is the second most common neurodegenerative disease [2]. In 2010, there were approximately 680,000 people over 45 years old with PD in the US in 2010 and this number is expected to rise to 1,238,000 in 2030 [3]. By the time PD becomes clinically apparent, more than 50% of dopaminergic neurons in the substantia nigra have been lost, with a corresponding 80% decline in striatal dopamine levels [4, 5]. Thus, early identification of disease is essential if neuroprotective therapies are to be implemented.

Diagnosis of PD currently relies on clinical examination. The current gold standard is based on motor signs and symptoms (bradykinesia, resting tremor, rigidity, postural reflex impairment) and response to dopaminergic drugs [6, 7]. In addition to the classic motor signs and symptoms, PD is well-recognized to also affect non-motor neural circuits [6, 8, 9] . However, the accuracy of PD diagnosis in practice is only around 80% [10, 11], implying that a large population with PD is undiagnosed or misdiagnosed [12]. Hence, identification of novel motor or non-motor markers of PD or improving the accuracy of currently available diagnostic tools is important, particularly in early disease. Noninvasive speech tests have been explored as a marker of disease [11, 13], since deterioration of speech is consistently observed in patients with PD [14–16]. Naranjo et al. [17, 18] previously showed that patients with PD could be identified with moderately high accuracy using acoustic features extracted from a speech test. In this study, we implemented machine learning methods, specifically Light [19] and Extreme Gradient Boosting [20], to significantly improve PD detection accuracy from acoustic features extracted from voice recordings.

## Methods

### Data

We utilized “Parkinson Dataset with Replicated Acoustic Features Data Set” that was donated to University of California Irvine Machine Learning repository by Naranjo, et al. [17] in April 2019. The publicly available data we used in this study were first presented by Goetz, et al. [21], and other than sex, individual-level descriptors are not publicly available. However, they reported that the dataset includes patients with early-stage PD not taking medication. A follow-up study [11] reported that PD duration was 5 years or less for all subjects, with a mean Unified Parkinson’s Disease Rating Scale (UPDRS) score of 19.6 (SD = 8.1). The dataset available to us [17] included 44 acoustic features extracted from voice recordings of 40 patients with PD and 40 controls. Recordings of a sustained phonation of the vowel /a/ for 5 s were repeated three times (three runs). Digital recordings were implemented at a 44.1 KHz sampling rate and 16 bits/sample [17].

The 44 acoustic features extracted from voice recordings comprised five categories: pitch and amplitude local perturbation, noise, special envelope, and nonlinear measures. Four pitch local features (jitter relative, jitter absolute, jitter RAP (relative absolute perturbation)), jitter PPQ (pitch perturbation quotient), and five amplitude perturbation measures (shimmer local, shimmer dB, APQ3 (3 point Amplitude Perturbation Quotient), APQ5 (5 pint Amplitude Perturbation Quotient), and APQ11(11-point Amplitude Perturbation Quotient)) were extracted using a waveform matching algorithm. As measures of relative level of noise in speech [17], five different variants of harmonic-to-noise ratio (HNR) corresponding to different frequency bandwidths (HNR05 [0–500 Hz], HNR15 [0–1500 Hz], HNR25 [0–2500 Hz], HNR35 [0–3500 Hz], HNR38 [0–3800 Hz]) [22]. Glottal-to-Noise Excitation Ratio (GNE), which quantifies the amount of voice excitation, was also calculated. Since PD is known to affect articulation [23], 13 Mel Frequency Cepstral Coefficients (MFCCs) associated with articular position and 13 Delta Coefficients as time dependent derivatives of MFCCs were also extracted. In addition, Recurrence Period Density Entropy (RPDE), Detrended Fluctuation Analysis (DFA), and Pitch Period Density Entropy (PPE) were also extracted as non-linear measures of voice recordings. Further details of the dataset can be found in Naranjo et al. [17].

### Features

**Fig. 1:**
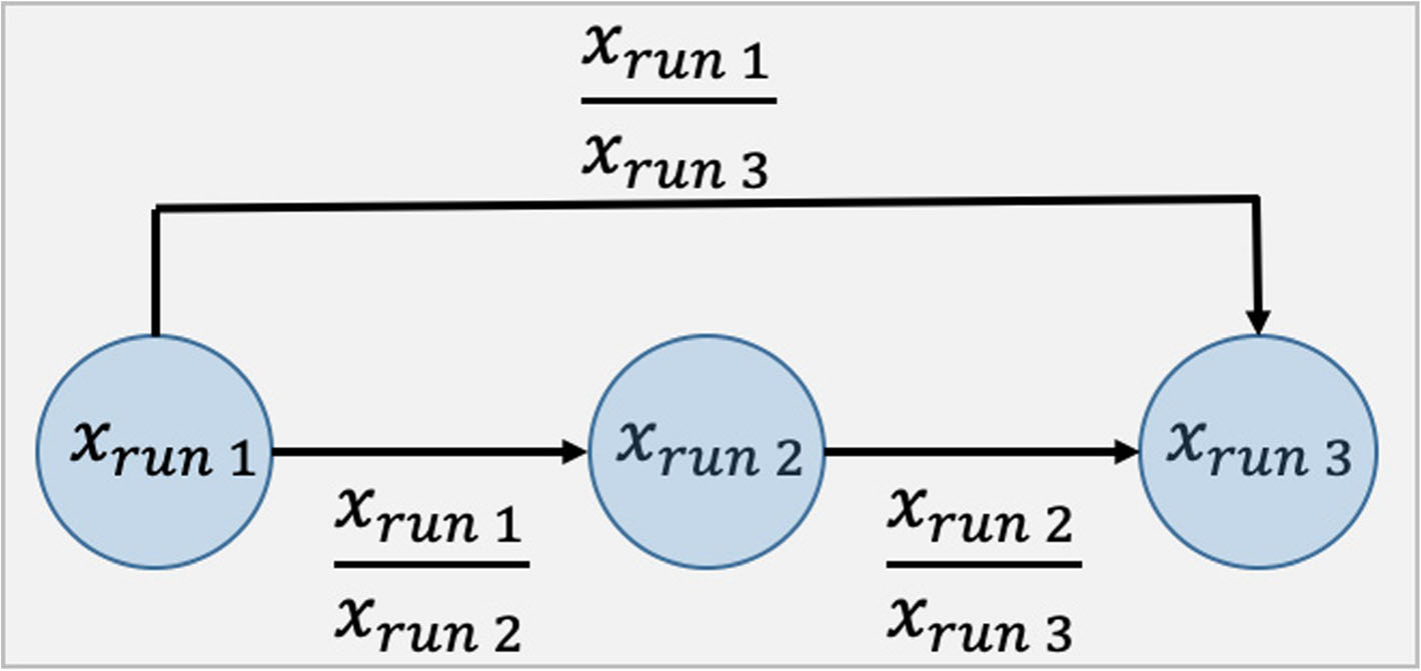
Acoustic features used in modeling

Speech deterioration is one of the motor symptoms of PD [14, 24–26]. Patients have reduced pitch variability compared to controls as well as reduced intra-individual variability [27, 28]. As described above, each acoustic feature was calculated three times for different runs of the speech test. Thus, in addition to testing the diagnostic accuracy of our analytic approach, we were also able to investigate intra-individual changes in response from different runs of the test. We considered acoustic features calculated for all three runs as individual predictors. Moreover, for a given acoustic feature, we created three artificial variables representing the change from one run to another (Fig. 1). Therefore, our feature set included 264 acoustic features and sex for 80 subjects.

### Classification

We implemented gradient boosting algorithms to distinguish between subjects with PD and controls. Gradient boosting is an ensemble machine learning consisting of several weak models (shallow decision trees rather than overfitting deep ones) and it can be used for both regression and classification problems [19, 20]. Because it uses weak classifiers, it is more robust against overfitting compared to a random forest, a similar method that allows overfitting of individual tree predictors [20, 29, 30]. In our work, we mainly implement 4-fold cross validation to identify any overfitting by randomly splitting data into four distinct folds. We also repeat this process multiple times and present average results. We considered two gradient boosting machines Extreme Gradient Boosting (XGB) and Light Gradient Boosting (LGB), and for comparison, the more traditional machine learning algorithms Random Forest (RF), Support Vector Machines (SVM), K-nearest neighborhood (KNN), Least Absolute Shrinkage and Selection Operator (LASSO) regression to implement regularization, and a statistical approach, Logistic Regression (LR).

### Variable importance analysis, feature selection, and re-classification

We first built the gradient boosting model using 265 features with four folds cross-validation and repeated this process 100 times. At each run, for each model built within 4-fold cross validation (4 × 100 models), we implemented a feature importance analysis that calculates the relative contribution of each feature to the corresponding model. A higher value of this metric for a specific feature implies it as a more important feature than another feature that has lower value of this metric [31]. By averaging the feature importance obtained from 400 individual models, we obtained a ranking of the 265 features. Next, we built new classification models with 4-fold cross-validation by incrementally adding the top 15 most important features selected from the previous step into the model with respect to their importance ranking. We repeated each of these steps 100 times to better estimate the effect of each feature on the model performance when they are introduced into the model. We then identified the step where the model performance started diminishing or stopped increasing. Finally, using the features introduced up to that specific step, we rebuilt gradient boosting models with 4-fold cross validation and report various performance metrics such as specificity, sensitivity, positive predictive value, accuracy, F1 score, and area under the receiver operating characteristics curve (AUC).

## Results

### Cohort

Our cohort included 40 subjects with PD (55% men) and 40 healthy controls (67.5% men). All subjects were over 50 years of age and the mean age and standard deviation (SD) for PD subjects and controls was 69.6 (SD 7.8) and 66.4 (SD 8.4), respectively. PD diagnosis required at least two of resting tremor, bradykinesia or rigidity [21], and no evidence for other forms of parkinsonism.

### Classification

We initially built the classification models with 4-fold cross-validation using the entire set of 265 predictors. We repeated each classification model 100 times by randomly splitting the data into four folds. Various classification performance metrics with their 95% Confidence Intervals (CI) are presented in Table 1. LGB provided the highest F1 score of 0.878 with 95% CI 0.871–0.884, and AUC of 0.951 (95%CI 0.946–0.955).

**Table 1:** Comparison of alternative machine learning methods. (LGB: Light Gradient Boosting, XGB: Extreme Gradient Boosting, LR: Logistic Regression, SVM: Support Vector Machines, RF: Random Forest, KNN: K-nearest Neighbor, LASSO: Least Absolute Shrinkage and Selection Operator)

Metrics		Accuracy Metrics with 95% CI					
	LGB	XGB	LR	SVM	RF	KNN	LASSO
F1	**0.839 [0.831–0.847]**	0.810 [0.802–0.819]	0.771 [0.762–0.780]	0.730 [0.721–0.739]	0.810 [0.800–0.819]	0.744 [0.735–0.753]	0.763 [0.755–0.7723]
AUC	**0.898 [0.892–0.905]**	0.891 [0.885–0.898]	0.839 [0.830–0.847	0.838 [0.830–0.846]	0.884 [0.876–0.892]	0.841 [0.834–0.848]	0.870 [0.863–0.877]
Accuracy	**0.841 [0.833–0.849]**	0.816 [0.809–0.823]	0.771 [0.762–0.780]	0.744 [0.735–0.752]	0.818 [0.810–0.826]	0.760 [0.752–0.768]	0.761 [0.753–0.769]
Sensitivity	**0.839 [0.827–0.850]**	0.801 [0.789–0.813]	0.777 [0.765–0.790]	0.704 [0.691–0.716]	0.795 [0.782–0.808]	0.712 [0.699–0.725]	0.782 [0.769–0.794]
Specificity	**0.844 [0.832–0.855]**	0.830 [0.819–0.841]	0.764 [0.750–0.778]	0.784 [0.771–0.798]	0.841 [0.831–0.852]	0.807 [0.796–0.818]	0.741 [0.729–0.754]
PPV	**0.853 [0.843–0.863]**	0.835 [0.825–0.845]	0.780 [0.769–0.791]	0.780 0.769–0.791]	0.844 [0.834–0.854]	0.796 [0.786–0.806]	0.762 [0.753–0.772]

### Variable importance analysis

**Fig. 2:**
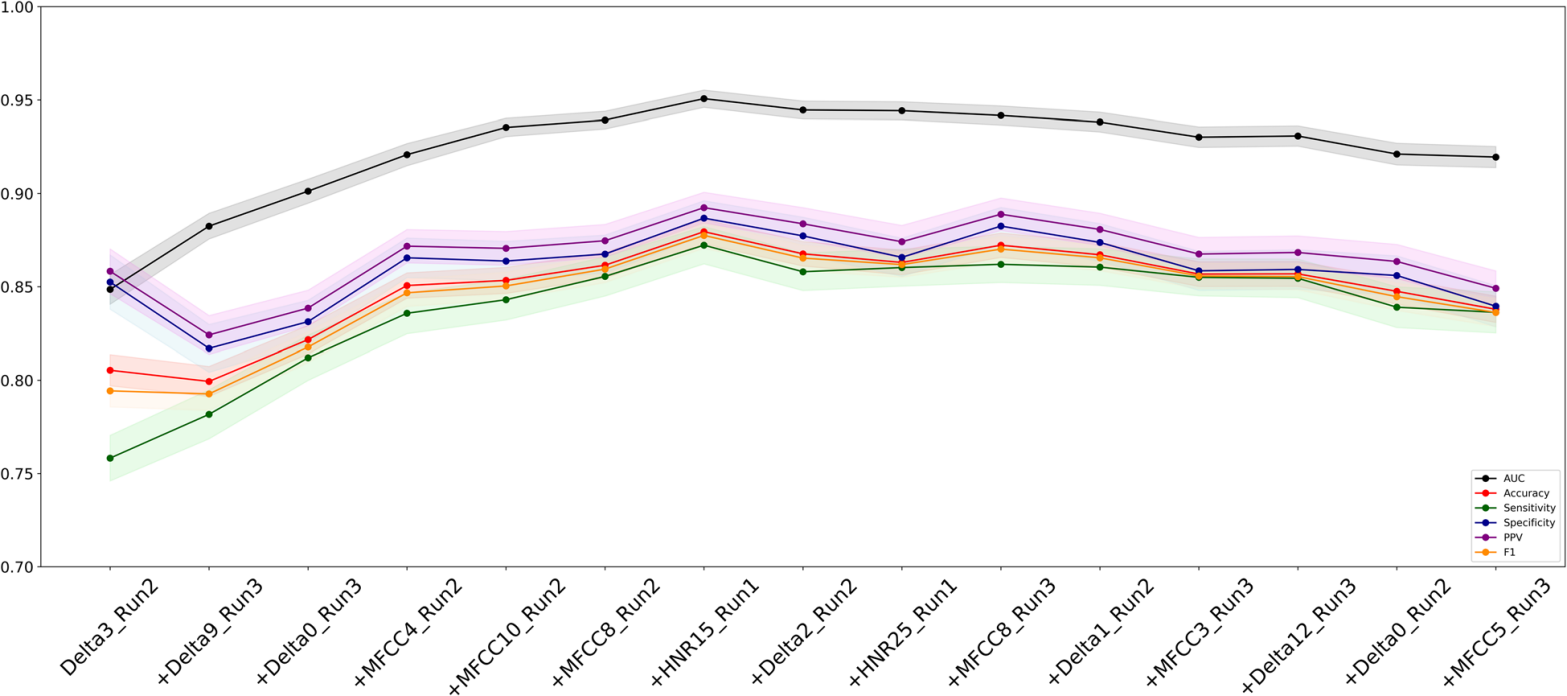
Feature selection and reclassification results for 4-fold cross validation using the LGB model

As described in the Methods section, using the total of 400 models obtained through 100 runs of 4-fold cross-validation, we obtained variable rankings based on their importance in classification in the LGB algorithm. The top 15 variables are shown on the x-axis of Fig. 2.

### Feature selection and re-classification

To obtain a compact model, we repeated our 4-fold classification strategy 15 times by incrementally introducing a new variable into the model based on the order of importance. Figure 2 summarizes the accuracy metrics with associated 95% CIs for each step of this re-classification.

Figure 2 shows that all accuracy metrics gradually increase (F1-score of 0.878 (95%CI 0.871–0.884), AUC of 0.951 (95% CI 0.946–0.955), Overall Accuracy of 0.880 (95% CI 0.873–0.886), Sensitivity of 0.872 (95% CI 0.862–0.882), Specificity of 0.887 (95% CI 0.877–0.896), Positive Predictive Value of 0.892 (95% CI 0.884–0.901)) in the first seven steps of the feature selection protocol and then slightly decline in following steps. In other words, after introducing the top seven variables - Delta3 (Run2), Delta9 (Run3), Delta0 (Run 3), MFCC4 (Run 2), MFCC10 (Run 2), MFCC8 (Run 2), and HNR15 (Run 1) - into the model, additional variables did not improve the classification accuracy. We further implemented a grid search by changing the learning rates and feature and bagging fraction to identify whether the performance could be improved. However, there was no significant difference in AUC values of models with different parameter settings.

Independent sample two-tail t-tests showed that the means of these top seven selected features significantly (*p* < 0.05) differed for PD cases and controls. To identify whether such differences exist for all three runs, we further implemented t-tests for those seven features for all runs. Our results showed the top seven acoustic features significantly (p < 0.05) differ for PD cases and controls across all three runs, however, the *p*-values are smaller for the runs that were listed in top seven features.

### Sensitivity analysis

The main reason for implementing 4-fold cross-validation in our work was to make our results comparable to the work of Naranjo et al. [17, 18], which is the original study utilizing these data. However, using the top seven variables, we also repeated our cross-validation on the compact model for 5- and 10- fold cross-validation for the light gradient boosting model and obtained F1-score of 0.879 (95%CI 0.872, 0.886) and 0.875 (0.867, 0.883), respectively.

The above models analyzed acoustic features from three runs as individual predictors. In a sensitivity analysis we explored whether using the average of acoustic features across the three runs might improve the model. This classification approach performed more poorly, with an F1-score of 0.819 (95% CI 0.812, 0.827) vs. 0.878 (95%CI 0.871, 0.884) for the individual predictor model.

## Discussion

We were able to accurately classify persons with Parkinson’s disease by analysis of voice recordings using machine learning. Acoustic features extracted from speech test recordings offer a potential application for computerized non-invasive diagnostic tools. The data we used in this study included 44 acoustic features generated separately for three runs of the same speech test task. In their original studies on the same data, Naranjo et al. [17, 18] proposed a statistical approach that treated the results of these runs as repeated measures. The Light Gradient Boosting model presented here outperformed the statistical approach in all metrics: AUC 0.951 vs. 0.879; sensitivity 0.872 vs 0.765; specificity 0.887 vs 0.792; precision 0.887 vs. 0.806; and overall accuracy 0.880 vs 0.779. Moreover, we could reach this level of accuracy using only seven features.

As reported above, Delta3 (Run2), Delta9 (Run3), Delta0 (Run 3), MFCC4 (Run 2), MFCC10 (Run 2), MFCC8 (Run 2), and HNR15 (Run 1) variables were the most important classifiers, and that these features were indeed significantly different for PD cases and controls across all three runs.

It is worth noting that only one of the seven acoustic variables obtained from the first run of the speech test was selected as a predictor in the final model. Four variables were from second run, and two from the third run. None of the variables representing changes from one run to another were selected as one of the top seven variables.

This study demonstrates that machine learning can assist clinicians in the accurate diagnosis of PD. Since the PD subjects in this study were in their early stages of disease, this approach may provide an opportunity for earlier diagnosis of PD. Future work should investigate whether such acoustic patterns exist during the prodromal phase of PD.

Our study has several limitations. The most important limitation is the small sample size. Despite the fact that our carefully designed cross-validation yielded very high accuracy, there is a need to repeat these analyses in a larger cohort. Moreover, the small sample size may also limit inferences of variable importance. Despite the fact that our model performed with high classification accuracy, the feature importance analysis must be cautiously interpreted since the ranks of importance may change when the study is repeated in a larger cohort. Additionally, all PD subjects in the dataset were drawn from a single study. External validation is needed to test the broader generalizability of our model. Another important limitation of our study is that our dataset includes only subjects with PD and controls. It is unclear whether our model can distinguish between subjects with PD and those with other diseases that can affect speech.

## Conclusions

Gradient boosting algorithms can be used to identify patients with Parkinson’s disease using a simple non-invasive speech test. Further studies are required to determine whether similar results can be obtained from records of normal conversation or phone calls. This approach could be used to screen large patient populations at different stages of Parkinson’s disease. The value of this approach to identify early prodromal PD remains to be determined.

## Data Availability

The data is available at UCI-Machine Learning Data Repository named “Parkinson Dataset with replicated acoustic features Data Set”.

## Abbreviations

PD: Parkinson’s Disease
UPDRS: Unified Parkinson’s Disease Rating Scale
SD: Standard Deviation
RAP: Relative Absolute Perturbation
PPQ: Pitch Perturbation Quotient
ABQ: Amplitude Perturbation Quotient
HNR: Harmonic-to-noise Ratio
MFCC: Mel Frequency Cepstral Coefficient
RPDE: Recurrence Period Density Entropy
DFA: Detrended Fluctuations Analysis
PPE: Pitch Period Density Entropy
XGB: Extreme Gradient Boosting
LGB: Light Gradient Boosting
RF: Random Forest
SVM: Support Vector Machines
KNN: K-nearest Neighborhood
LASSO: Least Absolute Shrinkage and Selection Operator
LR: Logistic Regression
AUC: Area Under the Receiver Operator Characteristics Curve
CI: Confidence Interval
